# Supplementary material for: Towards full-colour tunability of inorganic electrochromic devices using ultracompact fabry-perot nanocavities
Source: Nat Commun. 2020 Jan 16;11:302. doi: 10.1038/s41467-019-14194-y (PMC6965179; doi:10.1038/s41467-019-14194-y)
Supplement: Supplementary file 1 — Supplementary Information [file 41467_2019_14194_MOESM1_ESM.pdf]

Supplementary Information

Towards Full-colour Tunability of Inorganic Electrochromic Devices Using Ultracompact Fabry-Perot Nanocavities

Wang et al.

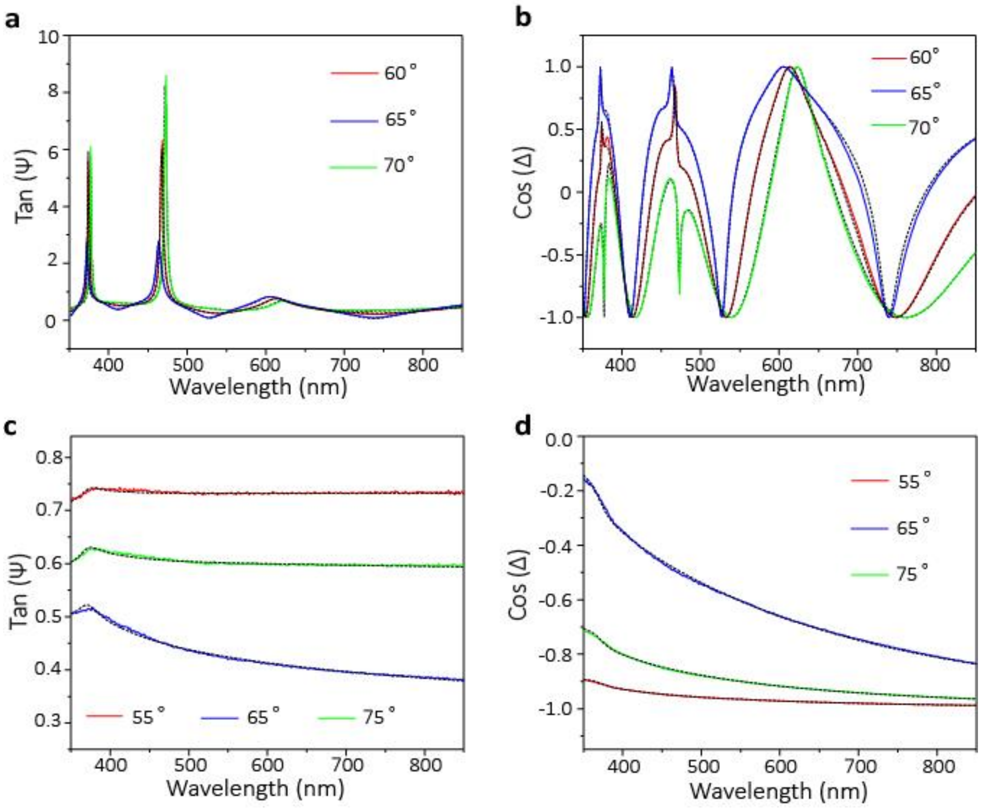

Supplementary Figure 1. Ellipsometry spectra for a, b) WO_3 and c, d) metal W under incident angles ranging from 60° to 70° with a step of 5° (experimental, coloured lines; simulation, black dash lines). Tan (ψ) and cos (Δ) represent the amplitude ratios of the parallel and perpendicular components, and the relative phase change, respectively.

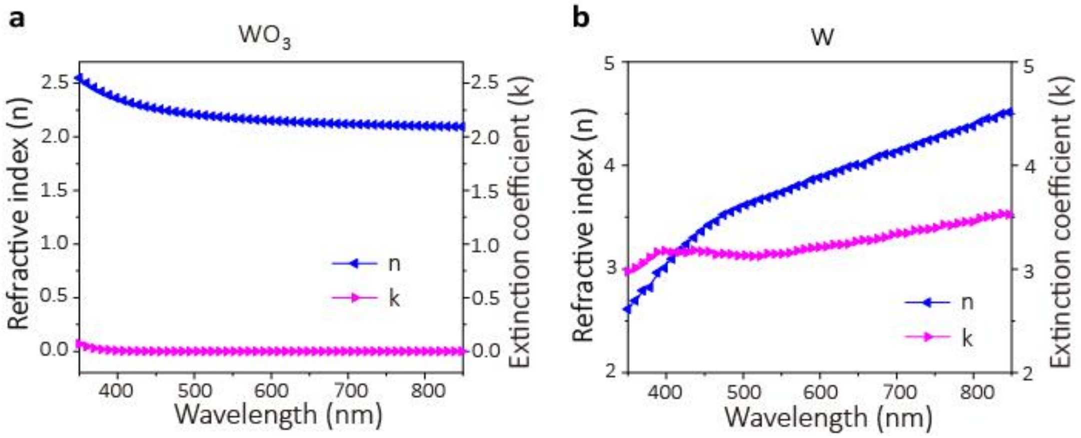

Supplementary Figure 2. Refractive index (n) and extinction coefficient (k) for a) WO_3 and b) metal W.

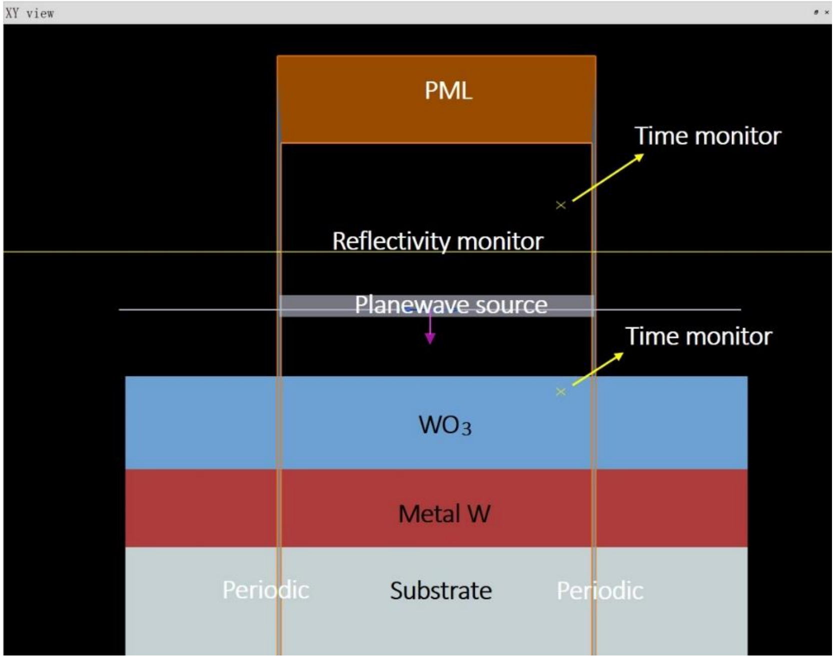

Supplementary Figure 3. FDTD simulation model for the F-P nanocavities on a substrate.

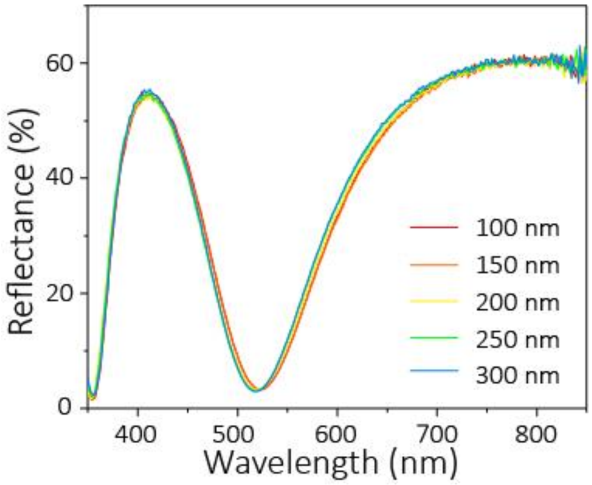

Supplementary Figure 4. Reflection spectra of the F-P nanocavities on a PET substrate, with the WO_3 layer at fixed thickness (163 nm) and the metallic W layer with different thicknesses ranging from 100 nm to 300 nm. All the curves coincide well, demonstrating that the thickness of the metallic W thin layer has no impact on the structural colours of the electrochromic electrodes.

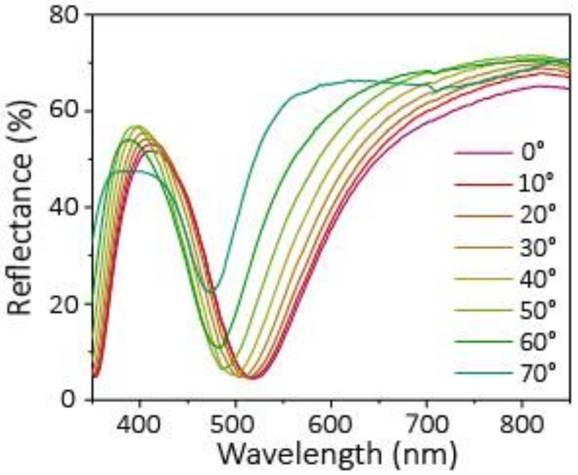

Supplementary Figure 5. Reflection spectra of the F-P nanocavity on the PET substrate with WO_3 at a thickness of 170 nm at different oblique incidence, with the angles ranging from 0° to 70°. It is noted that the position and intensity of the resonance maintains only small changes until the angle value is greater than 50°.

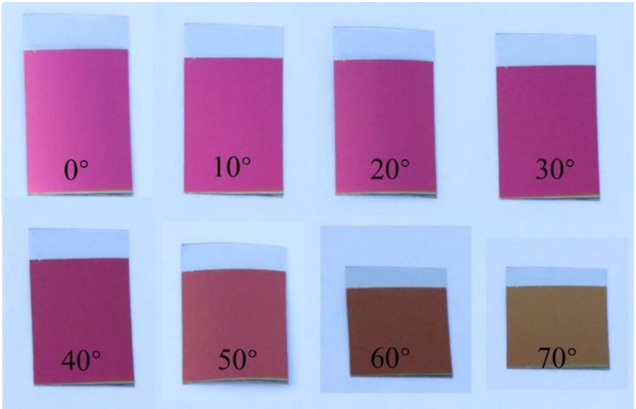

Supplementary Figure 6. Optical images of the fabricated F-P nanocavity on the PET substrate with WO_3 at a thickness of 170 nm, obtained from 0° to 70° with respect to the perpendicular view to the surface. It is clear that there is a small colour change even at large angles (up to 50°), validating good angle-independent reflectance behaviour.

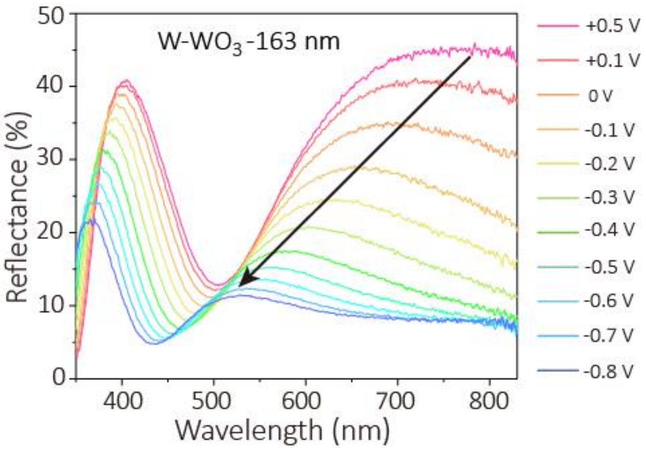

Supplementary Figure 7. The real-time reflection spectra of F-P nanocavity fabricated with the WO_3 layer thickness of 163 nm under varied driving potentials.

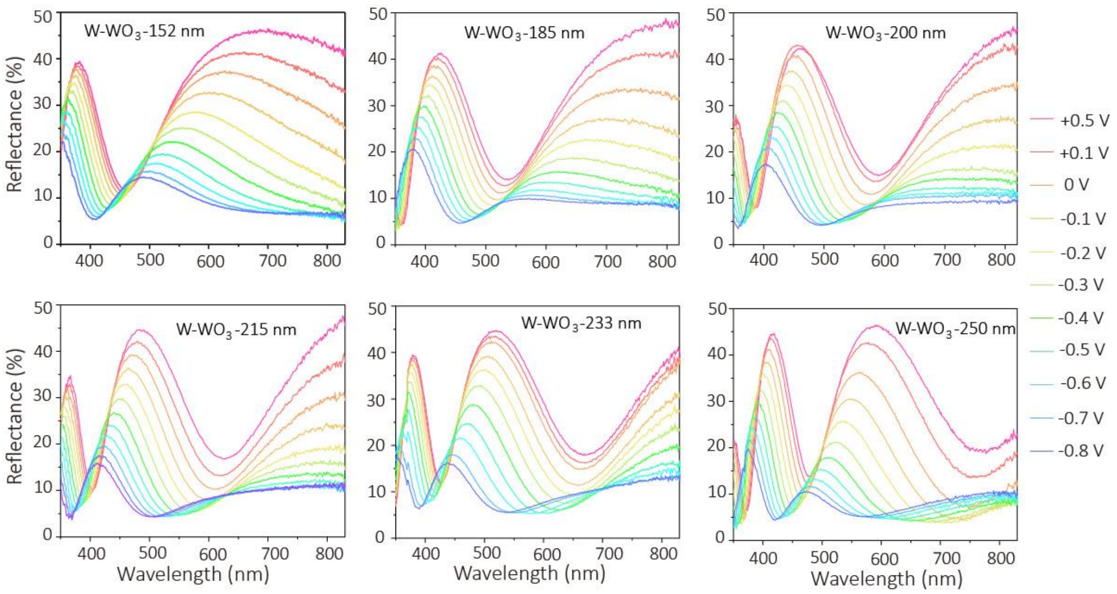

Supplementary Figure 8. The real-time reflection spectra of F-P nanocavities with the WO_3 layer thicknesses of 152 nm, 185 nm, 200 nm, 215 nm, 233 nm and 250 nm, respectively, under varied driving potentials.

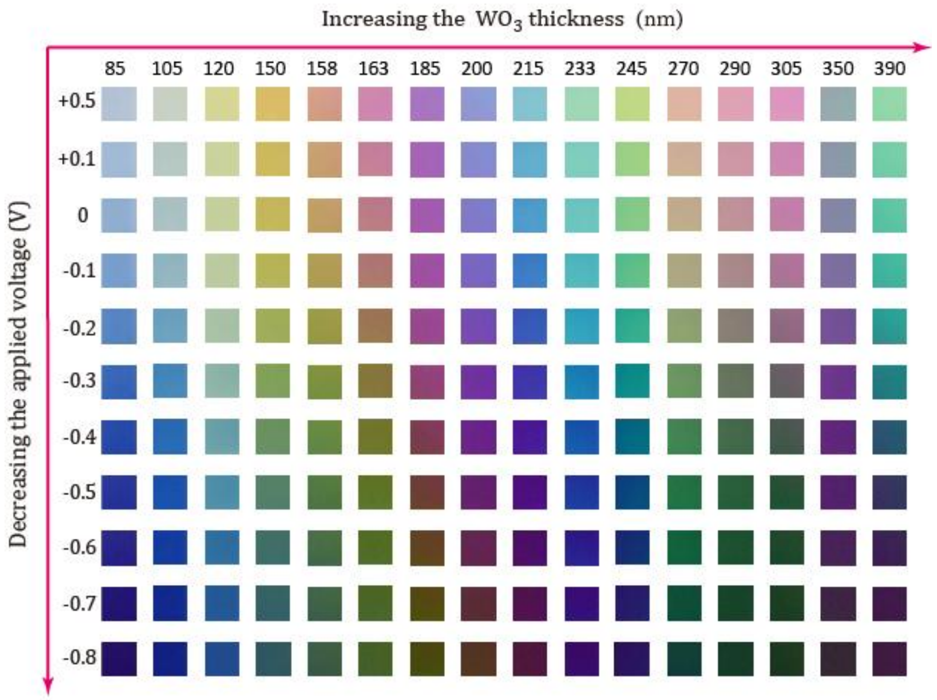

Supplementary Figure 9. A richer colour palette obtained from our F-P nanocavity-type electrochromic electrodes at different applied potentials.

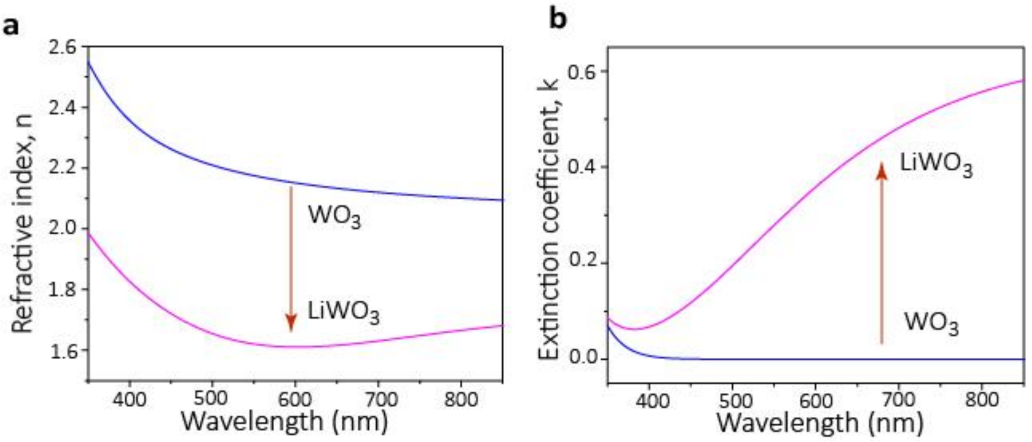

Supplementary Figure 10. The refractive indexes (a) and extinction coefficients (b) of the WO_3 film in bleached and coloured states.

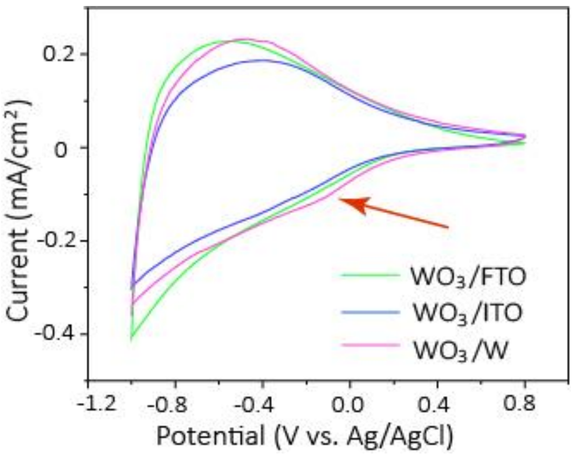

Supplementary Figure 11. Typical cyclic voltammograms of different electrochromic electrodes in 1 M $LiClO_4$/PC at an example scan rate of 10 mV s^{-1}. The three cyclic voltammograms curves are very accordant, illustrating that the metal W-covered F-P nanocavity-type electrochromic electrode maintains a comparable electrochemical performance with those of electrochromic electrodes applying traditional current collectors such as FTO and ITO.

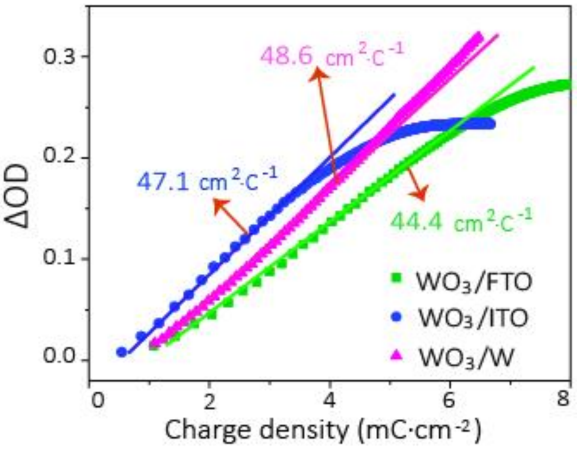

Supplementary Figure 12. Plot of optical density variation (ΔOD) as a function of charge density (Q) monitored at wavelength of 700 nm, which gives the colouration efficiency (CE) for different electrochromic electrodes. The CEs of the traditional FTO- and ITO-covered electrochromic electrodes are calculated to be 44.4 cm^2 C^{-1} and 47.1 cm^2 C^{-1}, respectively. The F-P nanocavity exhibits a CE of 48.6 $cm^2 \cdot C^{-1}$, which is slightly higher than those of the normal current collector-covered electrochromic electrodes.

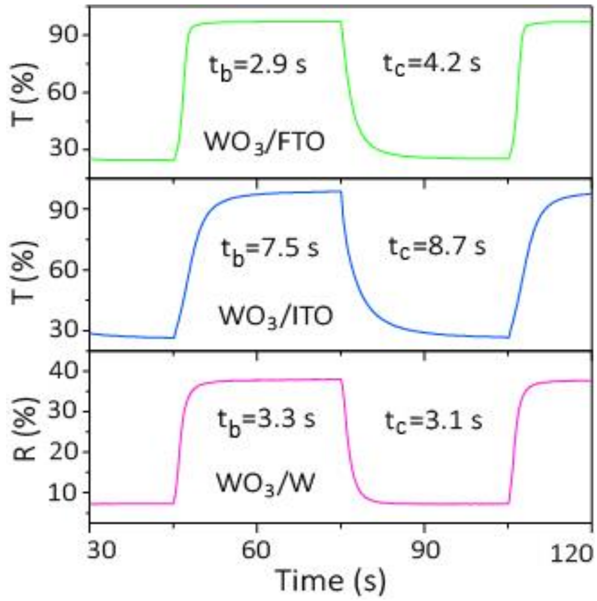

Supplementary Figure 13. Optical switching of different electrochromic electrodes, which determines the colouration (t_c) and bleaching (t_b) times. For traditional FTO- and ITO-covered electrochromic electrodes, the coloration times are found to be 2.9 and 7.5 s, and the bleaching times are 4.2 and 8.7 s, respectively. For the metal W-covered F-P nanocavity-type electrochromic electrode, the coloration time is 3.3 s, and the bleaching time is 3.1 s. Obviously, the switching speed of the metal W-covered F-P nanocavity-type electrochromic electrode is faster than that of the ITO-covered electrochromic electrode but is comparable to that of the FTO-covered electrochromic electrode.

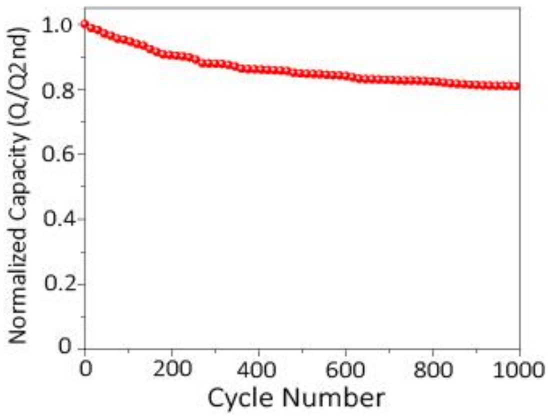

Supplementary Figure 14. Normalized charge capacity profiles of a W-covered F-P nanocavity-type electrochromic electrode over 1000 voltammetric cycles at 20 mV s^{-1}

between -0.8 and 0.5 V (vs. Ag/AgCl). Good cycling stability is demonstrated by a high capacity retention of 81% after 1000 cycles.

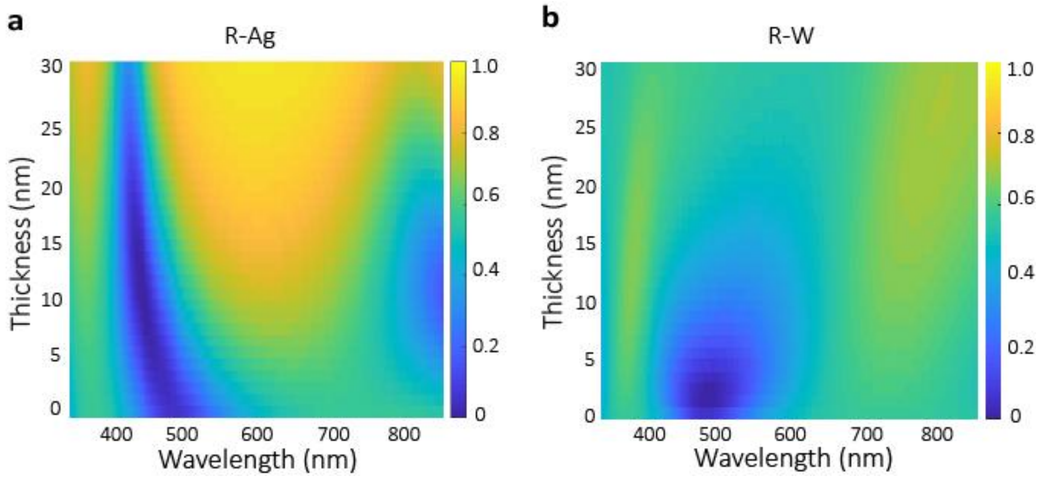

Supplementary Figure 15. Simulated reflectance intensity as a function of wavelength and top metal thickness (h) for a F-P nanocavity with the WO_3 layer thickness of 163 nm, with a) Ag top metal and b) W top metal, respectively. The results suggest that top metals have an influence on the intensity and peak of the reflectance of F-P nanocavities. In the case of the Ag top layer, the resonance in the reflection spectra becomes greater, while W top layer plays an opposite role.

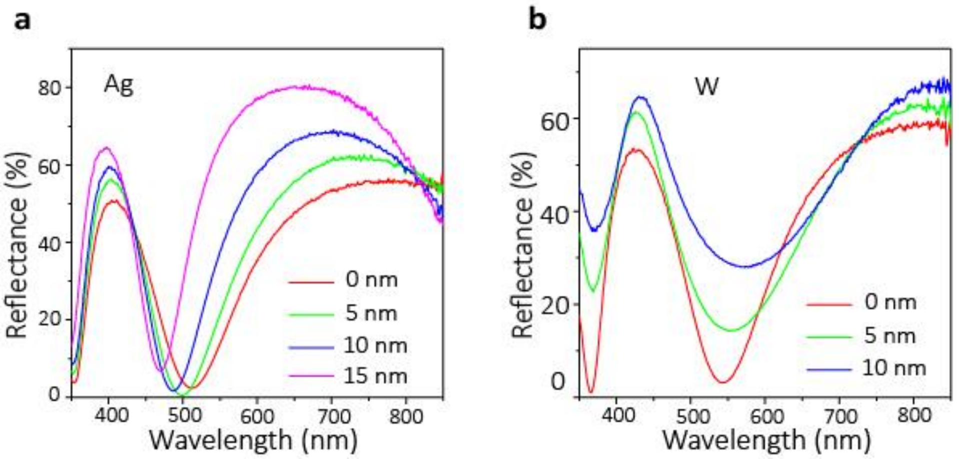

Supplementary Figure 16. The measured reflection spectra for F–P nanocavities with different thicknesses of Ag (a) and W (b) layers as top metal mirrors with unpolarized light at normal incidence.

Supplementary Table 1. Sputtering parameters for the fabricated films.

Films	Target	Gas flow (sccm)	Pressure (Pa)	Power (W)	Substrate rotating speed (rpm)
W	W	Ar: 20	0.3	200	20
WO_3	W	Ar: 71<br>O_2: 29	2.2	100	20
Ag	Ag	Ar: 20	0.3	50	20
NiO	NiO	Ar: 80<br>O_2: 12	2.0	100	20

Supplementary Table 2. Summary of the electrical conductivity, substrate surface area, and switching time results.

	FTO	ITO	W (100 nm)	W (200 nm)	W (300 nm)
substrate surface area (cm^2)	4.5	4.5	4.5	4.5	4.5
sheet resistance (Ω/□)	10	20	10	8	7
Switching times (t_b/t_c, s)	2.9/4.2	7.5/8/7	3.3/3.1	3.1/2.3	2.9/2.2

As can be seen from the table, the electrical conductivity of the substrates has a substantial effect on the electrochromic performances of the devices (particularly on switching times). Thus, given the same substrate surface area, the switching speed of the metal W-based F-P nanocavity-type electrochromic electrode is faster than the ITO-based electrochromic electrode but comparable to the FTO-based electrochromic electrode.

Supplementary Methods

Characterizations.

Field emission scanning electron microscopy (FE-SEM) analysis was performed on a FEI Quanta FEG field emission scanning electron microscope. XRD patterns of the prepared samples were recorded on a Bruker AXS D8 Advance X-ray diffractometer with a Cu Ka radiation target (40 V, 40 A). Tapping-mode atomic force microscopy (AFM; Bruker Instruments Dimension ion) with a silicon-tip cantilever (0.24 N m^{-1}) was used to characterize the top surface of the electrode.

Calculation of coloration efficiency.

$$CE = \Delta OD/\Delta Q = \log(T_b/T_c)/\Delta Q, \text{ or } CE = \Delta OD/\Delta Q = \log(R_b/R_c)/\Delta Q$$

Here ΔQ is the inserted charge that promotes the change (ΔOD) in the optical absorbance and T_b (R_b) and T_c (R_c) refer to the bleached and coloured transmittances (reflectance) at a certain wavelength, respectively. The CE can be accordingly evaluated from the slope of the plots of ΔOD versus ΔQ.

Calculation of optical constants for sputtering WO_3 and W.

The single-layer WO_3 and W deposited on silicon (100) substrates were probed via ellipsometry (Woollam M2000DI, 200–1700 nm) measurements. Under incident angles ranging from 60° to 70° with a step of 5°, the amplitude ratio of the parallel and perpendicular components of the reflected light (tan, ψ) and the relative phase change (cos, Δ), were obtained as a function of wavelength, as shown in Supplementary Figure 1. Data analysis was performed using WVASE32 software. Subsequently, the optical constants of the sputtering WO_3 and W layers were obtained by fitting the ellipsometry spectra using a quasi-static approach, as shown in Supplementary Figure 2.

Computational modelling of transmittance/reflectance spectra.

We use the characteristic matrix method derived from the basic principles of Maxwell's equations to model the transmittance/reflectance spectra of the F-P nanocavities. MATLAB is used to edit the calculation code. A detailed description of this process is provided by the following formula:[1]

$$\begin{bmatrix} B \\ C \end{bmatrix} = \begin{bmatrix} \cos\delta_1 & (i\sin\delta_1)/Y_1 \\ i\sin\delta_1 Y_1 & \cos\delta_1 \end{bmatrix} \begin{bmatrix} 1 \\ Y_2 \end{bmatrix}, \text{(S1)}$$

where B and C is the normalised electric and magnetic fields at the front interface and δ_1 is the phase thickness. This last property is defined as:

$$\delta_1 = \frac{2\pi N_1 d_1 \cos\theta_1}{\lambda}, \text{(S2)}$$

where N_1 and d_1 is the refractive index and the thickness of the first layer, respectively; θ_1 is the angle obtained from Snell's law (light is considered to have normal incidence for our study); and λ is the wavelength. Optical admittance values Y_1 and Y_2 are given by:

$$Y_1 = Y_0 N_1, \qquad \text{(S3)}$$

$$Y_2 = Y_0 N_2, \qquad \text{(S4)}$$

where Y_0 is the optical admittance in free space. The electric and magnetic components of Y_1 and Y_2 are equal.

The transmittance/reflectance spectra of the F-P nanocavities are thus calculated by equations (S5) and (S6), as shown below:

$$R = \left(\frac{Y_0 B - C}{Y_0 B + C}\right)\left(\frac{Y_0 B - C}{Y_0 B + C}\right)^*, \quad \text{(S5)}$$

$$T = \frac{4 N_2 {Y_0}^2}{(Y_0 B + C)(Y_0 B + C)^*}, \quad \text{(S6)}$$

where (.)* indicates a complex conjugate.

Finite difference time domain (FDTD) simulations

The simulation model is illustrated in Supplementary Figure 3. Periodic boundary conditions along the x- and y-axes are implemented for the simulation in a unit cell of 400 nm. Perfectly matched layers are set according to the propagation of electromagnetic waves (z-axis). Planewave sources are launched incident to the unit cell along the backwards z-direction. Two time-monitors are added to the simulation mode, and reflectance spectra are collected with a reflectivity monitor placed behind the radiation source. The complex refractive indexes (optical constants) of WO_3 and metal W for the simulation are based on the data illustrated in Supplementary Figure 2.

Supplementary Note 1. The material selection of metal layer.

According to common understanding, one of the greatest weaknesses of inorganic electrochromic materials is their monotonous colour changes. Thus, the ultimate goal of full-colour tunability for future electrochromic technology has been difficult to achieve with devices based on these typical materials. Our present work aims to broaden the colour versatility of inorganic electrochromic materials by introducing ultracompact Fabry-Perot (F-P) nanocavities into relative electrochromic devices. For a proposed F-P nanocavity-type electrochromic device, the metal layer is the central component, as this layer acts as a reflecting mirror for the optical interference that allows the generation of different colours. Generally, the choice of metal layer in our work is based on the following criteria.

(1) **Large dip depth**. As is well known, the depth of the resonance dip (that is, the difference in intensity between the dip minimum and the threshold) in a reflectance spectrum is an important indicator of resonance efficiency.[2] The greater the depth of this dip, the better the efficiency of resonance that will be achieved. Supplementary Figure17 shows the dependence of the depth of resonance dip on refractive index (n) and extinction factor (*k*) for the metal layers analysed in our work (for these measurements, 200-nm-thick WO_3 is set as the dielectric layer). The yellow region of the spectrum corresponds to a large dip depth, while the blue region indicates a small dip depth. As can be seen, the perceived (*n*, *k*) values for Al, Ag, Au and Cu are either fully or partly located in the blue region, indicating that the colour gamut was limited because a strong resonance could not be excited over the whole visible range. In contrast, the (*n*, *k*) values for Ni, Ti, Cr, V, Zn and W are all located in the yellow region, suggesting that a wide colour gamut is established by strong resonance behaviours operating across the entire visible spectrum.

(2) **Good stability**. We choose metal layers with good environmental and electrochemical stability.

(3) **Strong adhesion characteristics**. We choose metal layers that adhere well to the

substrate and the electrochromic layer.

According to the above criteria, W is selected as the ideal choice for the proposed F-P nanocavity-type electrochromic device.

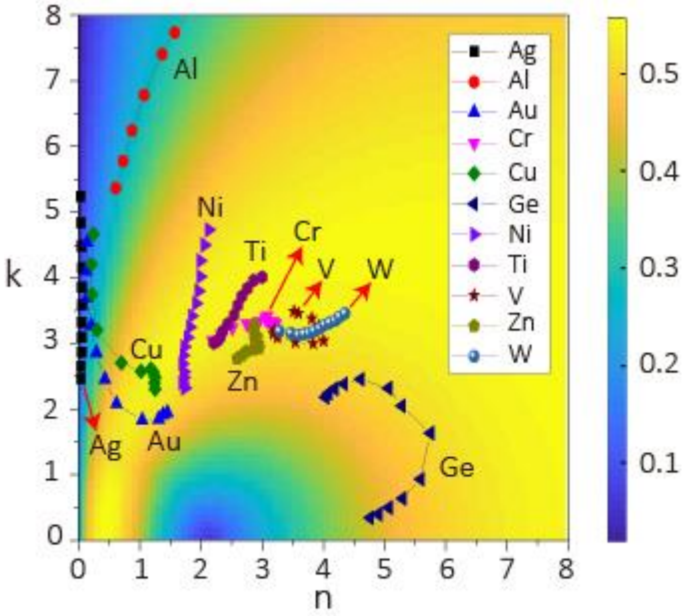

Supplementary Figure 17. The dependence of the depth of resonance dip on the refractive index (*h*) and extinction factor (*k*) of metal layers with different thicknesses. The parameters involved in the simulation are wavelength (400~800 nm) and WO_3 thicknesses (200 nm). The optical constant (*n*, *k*) of metal W is obtained by fitting the ellipsometry spectra using a quasi-static approach (Supplementary Figure 1, 2). The optical constants (*n*, *k*) of the other metals are all cited from the following website: http://refractiveindex.info (this source does not report an optical constant for metal W in the visible wavelength region).

Going further, the simulated reflectance spectra obtained from Al and Ag metal layers are given in Supplementary Figure 18. Only weak resonances are observed in the simulated reflectance spectra, with a maximum peak-to-valley fluctuation below 20%. This indicates that the reflected colours are only weakly perceptible in the cases of Ag and Al.

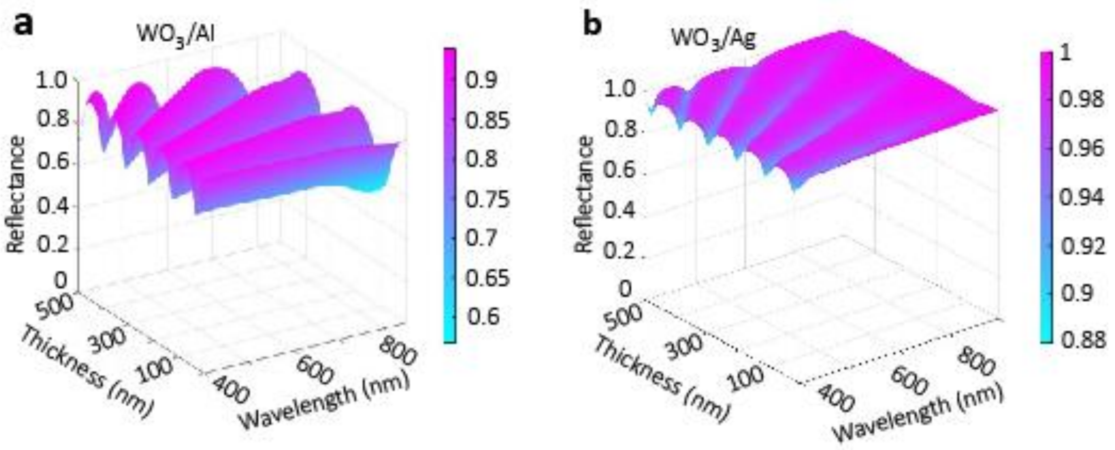

Supplementary Figure 18. Simulated reflectance spectra derived from Al (a) and Ag (b) metal layers.

Supplementary Note 2. NiO electrodes do not block the colours emitted by the WO_3 layer.

The optical image and transmittance spectra derived from the magnetron-sputtered NiO film are provided in Supplementary Figure 19, 20. As can be seen, the prepared films are almost transparent, with a high average transmittance of 85% over the whole visible region, demonstrating that they do not block the colour originating in the WO_3 layer. This result is consistent with the transmission spectra measured at different applied potentials for the electrochromic device consisting of two NiO electrodes paired with a transparent $LiClO_4$-based electrolyte. As shown in Supplementary Figure 20, the transmissions of the NiO electrodes and their corresponding devices remained nearly constant at the different applied potentials, suggesting that the NiO electrodes do not block the colours of the WO_3 layer.

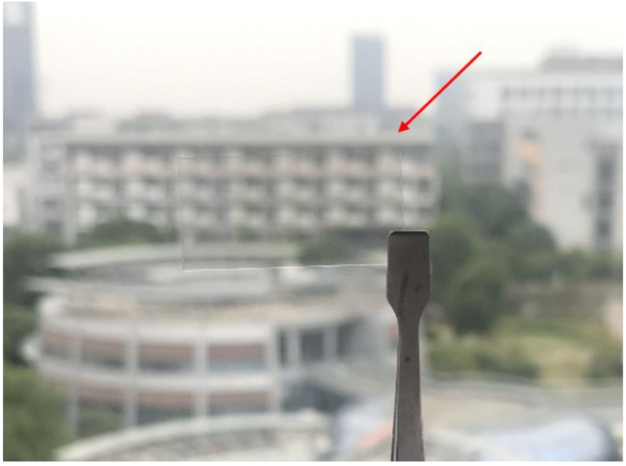

Supplementary Figure 19. An optical image of the NiO electrode (in the red rectangle), showing its high transmittance.

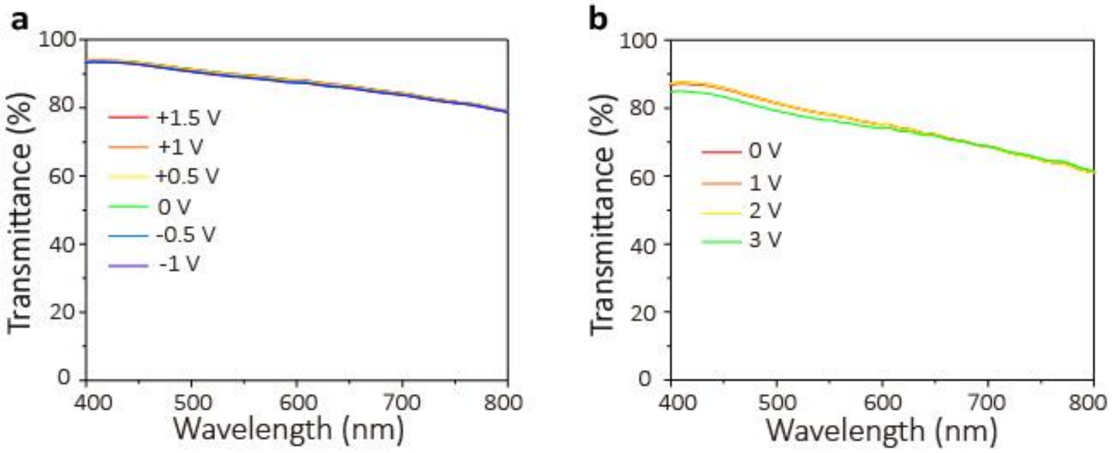

Supplementary Figure 20. The transmission spectra of the NiO electrodes with (a) the corresponding electrochromic devices at different applied potentials and (b) NiO electrodes paired with a transparent $LiClO_4$-based electrolyte. Significantly, during voltage application, the transmissions of the NiO electrodes and their corresponding devices remained almost constant, further suggesting that the NiO electrodes do not block the colours emitted by the WO_3 layer.

Supplementary Note 3.

For the experiments illustrated in Figure 2f, the reflectance valley of the F-P nanocavity-type electrode is red-shifted as the thickness of the WO_3 layer increased. These thickness-dependent shifts can be understood using the following equation:

$$\lambda_{DIV} = \frac{4n_1 d}{2k+1}\ (k = 0, 1, 2 \ldots)$$

where λ_{DIV} is the location of the wavelength intensity minima in the reflective spectrum (which has also been described as the destructive interference valley, DIV); d is the film thickness; and k is the DIV order. As can be clearly inferred from this equation, the wavelength positions of the DIV will be gradually red-shifted as the thickness of the tungsten oxide layer increases, thus resulting in different structural colors.

Supplementary References:

1. Wang, X. *et al.* High-temperature tolerance in WTi-Al_2O_3 cermet-based solar selective absorbing coatings with low thermal emissivity. *Nano Energy* **37**, 232-241, (2017).
2. Yue, W. *et al.* Color filters based on a nanoporous Al-AAO resonator featuring structure tolerant color saturation. *Opt. Express* **23**, 27474-27483, (2015).
